# Levels of circulating endothelial cells are low in idiopathic pulmonary fibrosis and are further reduced by anti-fibrotic treatments

**DOI:** 10.1186/s12916-015-0515-0

**Published:** 2015-11-09

**Authors:** Sara De Biasi, Stefania Cerri, Elena Bianchini, Lara Gibellini, Elisa Persiani, Gloria Montanari, Fabrizio Luppi, Cristiano Matteo Carbonelli, Luigi Zucchi, Marialuisa Bocchino, Alessandro Sanduzzi Zamparelli, Carlo Vancheri, Giacomo Sgalla, Luca Richeldi, Andrea Cossarizza

**Affiliations:** Department of Surgery, Medicine, Dentistry and Morphological Sciences, University of Modena and Reggio Emilia School of Medicine, via Campi, 287-41125, Modena, Italy; Department of Medical and Surgical Sciences for Children and Adults, University of Modena and Reggio Emilia, Modena, Italy; Department of Life Sciences, University of Modena and Reggio Emilia, Modena, Italy; Pulmonology Unit, Department of Cardiology, Thoracic and Vascular Surgery and Critical Care Medicine, IRCCS - Arcispedale Santa Maria Nuova, Reggio Emilia, Italy; Respiratory Medicine Section, Department of Clinical Medicine and Surgery, “Federico II” University of Naples, Naples, Italy; Regional Centre for Rare Lung Diseases, Department of Clinical and Experimental Medicine, University of Catania, Catania, Italy; Department of Respiratory Medicine, University of Southampton, Southampton, UK; Dipartimento Sperimentale Interaziendale, Campus San Lazzaro, University of Modena and Reggio Emilia, 42122 Reggio Emilia, Italy

**Keywords:** Circulating fibrocytes, Endothelial cells, Idiopathic pulmonary fibrosis, Nintedanib, Pirfenidone

## Abstract

**Background:**

It has been suggested that circulating fibrocytes and endothelial cells actively participate in the intense remodelling of the pulmonary vasculature in patients with idiopathic pulmonary fibrosis (IPF). Indeed, fibrotic areas exist that have fewer blood vessels, whereas adjacent non-fibrotic tissue is highly vascularized. The number of circulating endothelial cells (CEC) and endothelial progenitor cells (EPC) might reflect the balance between vascular injury and repair. Thus, fibrocytes as well as endothelial cells could potentially be used as biomarkers of disease progression and treatment outcome.

**Methods:**

Peripheral blood samples were collected from 67 patients with a multidisciplinary diagnosis of IPF and from 45 age-matched and sex-matched healthy volunteers. Buffy coat was isolated according to standard procedures and at least 20 million cells were stained with different monoclonal antibodies for the detection of CEC, EPC and circulating fibrocytes. For the detection of CEC and EPC, cells were stained with anti-CD45, anti-CD34, anti-CD133, anti-CD14, anti-CD309 and with the viability probe Far-Red LIVE/DEAD. For the detection of circulating fibrocytes, cells were first stained with LIVE/DEAD and the following monoclonal antibodies: anti-CD3, anti-CD19, anti-CD45, anti-CD34 and anti-CD14, then cells were fixed, permeabilized and stained with fluorochrome-conjugated anti-collagen I monoclonal antibodies.

**Results:**

Patients with IPF displayed almost undetectable levels of circulating fibrocytes, low levels of CEC, and normal levels of EPC. Patients treated with nintedanib displayed higher levels of CEC, but lower levels of endothelial cells expressing CD309 (the type II receptor for vascular endothelial growth factor). Treatment with both nintedanib and pirfenidone reduced the percentage of CEC and circulating fibrocytes.

**Conclusions:**

Levels of CEC were reduced in patients with IPF as compared to healthy individuals. The anti-fibrotic treatments nintedanib and pirfenidone further reduced CEC levels. These findings might help explain the mechanism of action of these drugs and should be explored as predictive biomarkers in IPF.

## Background

Idiopathic pulmonary fibrosis (IPF) is a chronic, progressive, irreversible and ultimately lethal lung disease of unknown cause and unclear pathogenic mechanisms, characterized by myofibroblast accumulation and lung scarring [1, 2]. At present, there are no reliable clinical parameters or non-invasive biomarkers predicting the clinical course of IPF [3]. A growing body of evidence indicates that the disease can result from the abnormal behaviour of the alveolar epithelial cells, which provokes the migration, proliferation and activation of mesenchymal cells. This results in the formation of fibroblast and myofibroblast foci secreting exaggerated amounts of extracellular matrix molecules, with the subsequent destruction of the lung architecture [4]. It has been hypothesized that an extra-pulmonary source of fibroblast/myofibroblasts exists, which likely has a bone marrow origin and can be detected in the blood [5].

In 1994, using state-of-the-art techniques, circulating fibrocytes were identified as cells that exit the blood stream, migrate into wounds and contribute to wound repair [6]. Fibrocytes are spindle-shaped, bone marrow-derived mesenchymal progenitor cells that co-express a variety of cell surface markers related to leukocytes, hematopoietic progenitor cells and fibroblasts. They express a variety of mesenchymal markers, including collagen I, as well as the common leukocyte marker CD45 and the hematopoietic stem cell marker CD34. They do not express T cell markers (CD3, CD4 and CD8), B cell markers (CD19) or myeloid markers (CD14) [7]. It has been shown that, in healthy donors, they can represent up to 1 % of circulating nucleated cells [8–11] and can express chemokine receptors such as CXCR4 and CCR7; they have been found in a variety of tissues under both physiological and pathological states [9, 12]. However, scanty data exist on the fine characterization of these circulating cells, whose relative rarity in blood obviously represents an obstacle to their precise analysis.

The biological axis CXCL12/CXCR4 could be involved in mediating the contribution of fibrocytes to pulmonary fibrosis [10]. Indeed, the high expression of CXCL12 in lung injury creates a chemokine gradient for CXCR4+ fibrocytes, which can be released from the bone marrow and recruited to the lungs [13]. Once they extravasate and enter the target tissue, fibrocytes can differentiate into fibroblasts and myofibroblasts [14]. So, it has been supposed that circulating fibrocytes might contribute to the intense remodelling of the pulmonary vasculature in patients with IPF, or at least represent a biomarker of disease activity [15].

Multiple mechanisms play a role in IPF pathogenesis, including abnormal vascular repair and remodelling [16]. During IPF, fibrogenesis is strongly associated with abnormal vascular remodelling [17]. Indeed, there is a body of evidence suggesting that the impairment of re-endothelization mechanisms after alveolar injury may lead to the destruction of lung architecture, and consequently trigger fibrosis [18]. Failure of re-endothelization may induce loss of the alveolar-capillary integrity, which might be the point after which fibrosis becomes inevitable [16]. Fibrotic areas have few blood vessels, whereas adjacent non-fibrotic tissue is highly vascularized [19]. There are almost no capillaries within the fibroblastic foci, indicating that the fibrotic process in IPF does not need neovascularization [20]. In this regard, it has been suggested that the respective abundance of circulating endothelial cells (CEC) and endothelial progenitor cells (EPC) might reflect the balance between vascular injury/repair and potentially serve as biomarkers of the disease [17]. Few data on CEC or EPC exist from patients with IPF.

With the aim of clarifying whether CEC and their precursors and circulating fibrocytes are altered in IPF, and to understand whether these cells may be used as biomarkers, we studied such cells in a cohort of Italian patients with IPF, some of whom were longitudinally followed. We used an innovative methodological approach, based upon sophisticated techniques that employ acoustic, multiparametric flow cytometry that allows a precise and fine analysis of these rare cell types.

## Methods

### Patients

All incident and prevalent patients with IPF from six Italian centres (Modena, Reggio Emilia, Bologna, Siena, Napoli and Catania) were deemed eligible for this study. All patients fulfilled 2011 American Thoracic Society/European Respiratory Society/Japanese Respiratory Society/Latin American Thoracic Association guideline diagnostic criteria [21]. Complete medical history and lung function tests were acquired at enrolment. Six-month follow-up visits and lung function tests were scheduled for up to 2 years. Blood samples for the analysis of circulating fibrocytes and endothelial cells were obtained at enrolment and during follow-up visits.

The study has been approved by the Local Ethical Committee (Modena, Number of practice 31/12), and written informed consent was obtained from each patient.

Among the patients with IPF, 18 were treated with pirfenidone, 13 with nintedanib, and 26 were untreated. Patient characteristics are reported in Table 1.

**Table 1:** Patients’ characteristics

	Number	Percentage	Median	IQR
Gender				
Male	53			
Female	14			
Age (years)			74	68.5–77.0
Time from diagnosis (years)			3	2.0–4.5
Smoking history				
Non smoker	19			
Smoker or former smoker	41			
Forced vital capacity (% predicted)		75.0		56.75–93.0
DLCO (% predicted)		41.0		34.0–60.0
GAP stage (%)				
I		32.70		
II		53.10		
III		14.30		
Treatment				
Pirfenidone	18			
Nintedanib	13			
Untreated	26			

*DLCO* Diffusing capacity of the lungs for CO_2_, *GAP* Gender, Age and Physiology Index, *IQR* interquartile range

### Blood collection and cell analysis

Thirty millilitres of blood were collected through a venous drawing in EDTA tubes. The first 3 mL of blood from the venipuncture were not used for cell analysis, because of the contaminating presence of endothelial cells derived from the vessel wall. Buffy coat was then prepared according to standard procedures, and cells were stained with different monoclonal antibodies (mAbs) for the detection of CEC, EPC and circulating fibrocytes. For the detection of CEC and EPC a minimum of 10 millions cells were stained with anti-CD45 PE (eBioscience, San Diego, CA, USA), anti-CD34 PC7 (Beckman Coulter, Hieleah, FL, USA), anti-CD133 APC (Miltenyi GmbH, Bergisch Gladbach, Germany), anti-CD14 APC-VIO770 (Miltenyi), anti-CD309 FITC (R&D Systems, Minneapolis, MN, USA) and viability probe Far-Red LIVE/DEAD.

For the detection of circulating fibrocytes a minimum of 20 million cells were stained with Red Fixable LIVE/DEAD probe (Thermo Fisher,Eugene, OR, USA ) and the following surface mAbs: anti-CD3 PE-CY 5.5 (Becton Dickinson, San José, CA, USA), anti-CD19 PE-CY 5.5 (Becton Dickinson), anti-CD45 PE (eBioscience), anti-CD34 PC7 (Beckman Coulter), anti-CD14 APC-VIO770 (Miltenyi) and anti-CXCR4 APC (Becton Dickinson). Cells were fixed and permeabilized using Cytofix/Cytoperm buffer set (Becton Dickinson) and stained with directly conjugated mAb anti-collagen I FITC (Merck Millipore, Billerica, MA, USA). Tables 2 and 3 report the mAbs used and the relative fluorochromes.

**Table 2:** Table summarizing the excitation sources and fluorescence emissions used for the detection of circulating endothelial cells and their precursors

Monoclonal antibody (mouse anti-human)	Directly conjugated fluorochrome	Excitation/emission (nm)
Anti-CD34	PC7	488/750
Anti-CD45	PE	488/530
Anti-CD133	APC	637/660
Anti-CD309	FITC	488/519
Anti-CD14	APCVio770	637/785
LIVE/DEAD	Far Red	637/>665

**Table 3:** Table summarizing the excitation sources and fluorescence emission used for the detection of circulating fibrocytes

Monoclonal antibody (mouse anti-human)	Directly conjugated fluorochrome	Excitation/emission (nm)
Anti-CD34	PC7	488/750
Anti-CD45	PE	488/530
Anti-CXCR4	APC	637/660
Anti-collagen I	FITC	488/519
Anti-CD19	PE-Cy5.5	488/690
Anti-CD3	PE-Cy5.5	488/690
Anti-CD14	APCVio770	637/785
LIVE/DEAD	Red Fixable	488/615

### Acquisition of samples

For phenotype analysis, cells were acquired using a 14-colour 4-laser high-speed Attune NxT flow cytometer (Thermo Fisher). Single staining and fluorescence minus one (FMO) controls were performed for all panels to set proper compensation and define positive signals [22]. In order to identify rare cells like human peripheral CEC, EPC or circulating fibrocytes, it was mandatory to acquire a huge number of cells [23], that is, of the order of several million per sample. Thus, for the phenotypic analysis, we used a novel acoustic flow cytometer able to align cells in the flow chamber using ultrasound, acquiring up to 35,000 cells per second. This was crucial to obtain the number of cells required for a correct statistical analysis, which was typically >10 million. Starting from a buffy coat, we were therefore able to clearly identify CEC, EPC or fibrocytes among peripheral blood cells.

### Statistical analyses

Data were analysed by FlowJo 9.8.5 and GraphPad 6.0 software, using the Wilcoxon T test and non-parametric analysis of variance test (Kruskal–Wallis test).

## Results and discussion

### Detection of fibrocytes, CEC and EPC requires the analysis of a large number of cells

**Fig. 1:**
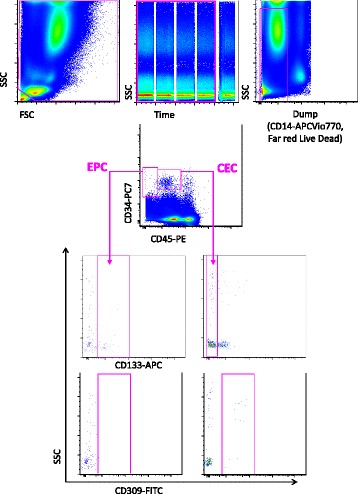
Gating strategy for the identification of circulating endothelial cells (*CEC*) and endothelial progenitor cells (*EPC*). Debris, monocytes and dead cells were excluded by the use of an electronic gate and the dump channel, containing cells identified by mAbs against CD14 and a viability marker, i.e. LIVE/DEAD. CEC and EPC were identified on the basis of the expression of CD34, CD45 and CD133: CEC were defined as CD45dim, CD34+ and CD133− while EPC were defined as CD45−, CD34+ and CD133+. The expression of CD309 (VEGFR-2, KDR) was detected among EPC and CEC. *FSC* forward scatter, *SSC* side scatter

The cytometric approach that we used in this investigation was different from that of previous studies, which, mainly for technical reasons, could only analyse a relatively low number of events. The high number of cells that we could acquire and analyse, along with the use of Poisson statistics, allowed a correct interpretation of the data [23]. As shown in Fig. 1, which reports a representative analysis of CEC and EPC, cells were first selected according to physical parameters; debris and aggregates were then removed according to the forward scatter (FSC)-A versus FSC-H dot plot. In this population, dead cells and monocytes were removed using a ‘dump’ channel. The parameter ‘time’ shown in the middle upper panel was used to monitor the stability of the flow cytometric high-speed acquisition of events. CEC and EPC were identified on the basis of the expression of CD34, CD45 and CD133: CEC were defined as CD45dim, CD34+ and CD133− while EPC were defined as CD45−, CD34+ and CD133+ [24]. The parental population was represented by peripheral blood mononuclear cells that were alive (i.e. negative to LIVE/DEAD staining) and negative for CD14. Expression of CD309 (i.e. the type II receptor for the vascular endothelial growth factor, VEGFR-2, also named KDR) was detected among EPC and CEC.

**Fig. 2:**
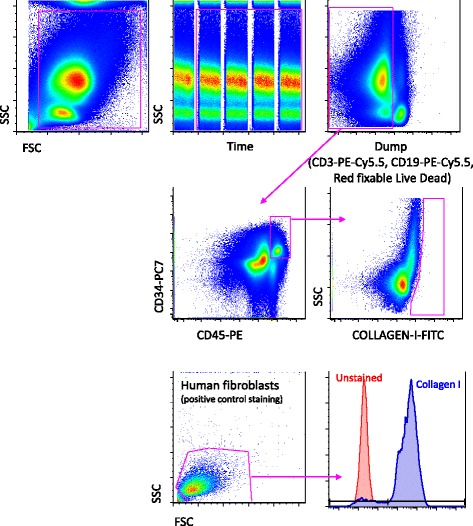
Gating strategy for the identification of circulating collagen I+ cells, i.e. fibrocytes. Debris, B cells, monocytes and dead cells were removed by an electronic gate as described in the legend to Fig. 1. Circulating fibrocytes were identified as CD34+, CD45+ and collagen I+. The expression of CXCR4 was then evaluated among circulating fibrocytes. Lower panels, referred to cultured human fibroblasts, represents a positive control of collagen I staining (>95 % cells were positive). *FSC* forward scatter, *SSC* side scatter

The gating strategy used for the identification of circulating fibrocytes involved the exclusion of aggregates (by using an FSC-A versus FSH-H dot plot). In this population, T lymphocytes, B lymphocytes and dead cells were excluded. In this pure population, circulating fibrocytes were defined as CD34+, CD45+ and collagen I+ cells (Fig. 2). The FMO approach was also used to detect positive cells. Moreover, we could search for the presence of CXCR4 circulating fibrocytes (see below).

### Low levels of circulating fibrocytes in patients with idiopathic pulmonary fibrosis

By using a sophisticated multiparametric analysis on a very sensitive flow cytometer, we could study and precisely quantify the presence of fibrocytes in patients with IPF. It is of note that, in order to avoid a possible unspecific staining due to a secondary antibody, we used a directly conjugated mAb recognizing collagen I and freshly isolated peripheral blood cells (Fig. 2).

In patients with IPF, the proportion of fibrocytes was below 1 % in almost all samples (see a representative example in Fig. 2, middle right panel). This was also the case in healthy controls (not shown). This result is in contrast with previous studies performed on freeze-thawed, fixed, permeabilized peripheral blood, which claim that the percentage of fibrocytes in acute IPF can be as high as 20 % [25]. This observation has been argued by others, because samples were not optimally used [26], and the number of events was likely too low to reach statistical significance. Moreover, no functional analysis have been performed on purified populations of fibrocytes isolated from blood to demonstrate their lineage. In our study, we have not confirmed the previously reported high levels of fibrocytes in blood from patients with IPF. Moreover, because it is almost impossible to sort and perform functional analysis of such cells (i.e., cells that are not viable, because their cytometric identification through the identification of collagen I requires permeabilization of plasma membrane and cell fixation), further studies are needed to clarify the meaning of collagen I+ cells, which are at present defined as fibrocytes, in peripheral blood.

### Patients with idiopathic pulmonary fibrosis displayed low levels of circulating endothelial cells

**Fig. 3:**
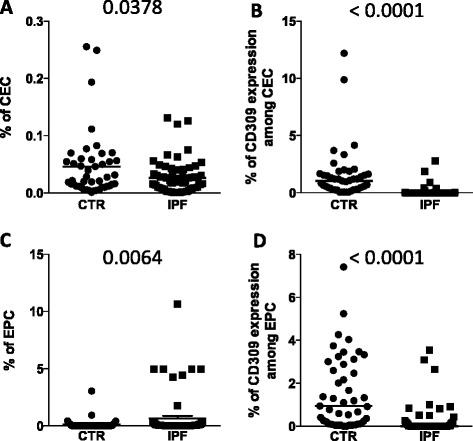
Percentages of circulating endothelial cells (*CEC*) and endothelial progenitor cells (*EPC*) in patients with idiopathic pulmonary fibrosis (*IPF*) and healthy participants. Scatter plots and median (line) indicating (**a**) the percentages of CEC; (**b**) the amount of CD309 among CEC; (**c**) the percentage of EPC; (**d**) the amount of CD309 among EPC in healthy donors (*CTR*) and patients (*IPF*). *P*-values, calculated using a Mann–Whitney test, are indicated in the figure

Patients with IPF displayed low levels of CEC (Fig. 3a), along with a significantly lower amount of CEC expressing CD309 (Fig. 3b), as compared to controls. They also displayed a slightly higher percentage of EPC (Fig. 3c), which, however displayed a lower expression of CD309 (Fig. 3c) than those of healthy participants. It is noteworthy that six out of seven of the patients with the highest levels of EPC were untreated.

The decrease in CD309 expression could be due to different factors, ranging from the progression of the disease per se in untreated patients to the pharmacological effect of pirfenidone and nintedanib, which can decrease the expression of CD309/VEGF-R, altering the VEGF–VEGFR axis [27]. For instance, nintedanib binds to the intracellular ATP-binding pocket of fibroblast growth factor (FGF) receptors, platelet-derived growth factor (PDGF) receptors and VEGFRs, blocking the autophosphorylation of these receptors and the downstream signalling cascades (reviewed in [28]). Alternatively, it could be hypothesized that CEC are able to home into injured tissue to participate in lung re-endothelization, and this phenomenon diminishes their number in peripheral blood.

### Patients with idiopathic pulmonary fibrosis treated with nintedanib have a higher level of circulating endothelial cells, but a lower number of endothelial cells expressing CD309

**Fig. 4:**
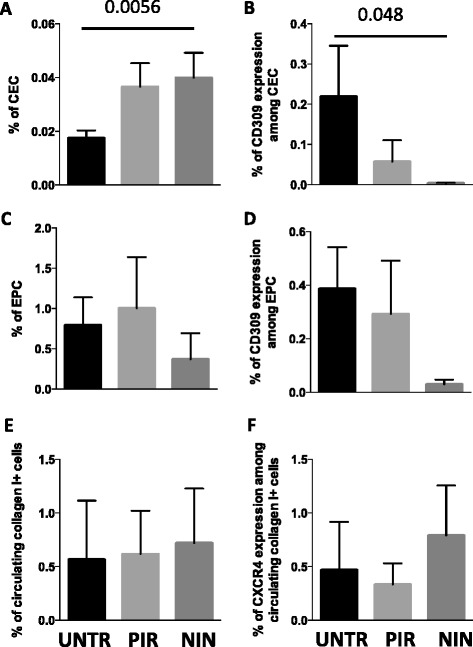
Percentages of circulating endothelial cells (*CEC*), endothelial progenitor cells (*EPC*) and circulating fibrocytes in patients with idiopathic pulmonary fibrosis treated with different therapies. Bar plots with error bars (mean + standard error of the mean) indicating (**a**) the percentage of CEC; (**b**) the amount of CD309 among CEC; (**c**) the percentage of EPC; (**d**) the percentage of EPC expressing CD309; (**e**) circulating collagen I+ cells; (**f**) the amount of CXCR4 among fibrocytes in untreated patients (*UNTR*) and in patients treated with pirfenidone (*PIR*) or nintedanib (*NIN*). *P*-values, calculated using a Kruskal–Wallis test, only significant values are displayed

We then compared the levels of endothelial cell populations and collagen I+ cells in untreated and treated patients with IPF. Untreated patients displayed lower levels of CEC than patients treated with nintedanib or pirfenidone (Fig. 4a); treated patients also displayed lower levels of CEC expressing CD309 (Fig. 4b). Among these three groups of patients with IPF, we did not find statistically significant differences in EPC population (Fig. 4c), although the percentage of EPC expressing CD309 was lower in patients treated with nintedanib (Fig. 4d). The percentage of circulating collagen I+ cells, defined as fibrocytes (Fig. 4e), and of fibrocytes expressing CXCR4 (Fig. 4f) was similar between untreated and treated patients.

### Nintedanib and pirfenidone reduce the percentage of circulating endothelial cells and circulating fibrocytes (collagen I+ cells) after 6 months of treatment

We analysed the percentage and the phenotype of CEC, EPC and circulating fibrocytes in 12 patients before and after 6 months of anti-fibrotic treatment. It has to be underlined that the detection and quantification of circulating fibrocytes, that is, cells that express collagen I, is quite problematic for several reasons, starting with their extremely low number. Furthermore, to be extremely rigorous, we cannot exclude the possibility that some CD14+ cells that express CD34 (or that just bind unspecifically to the anti-CD34 mAb by Fc receptors) could express collagen I, and thus this population could become an artefact of the analysis. In any case, given that this hypothesis is quite unlikely and we performed all possible quality control measures, we clearly show that collagen I+ cells decrease significantly after therapy.

**Fig. 5:**
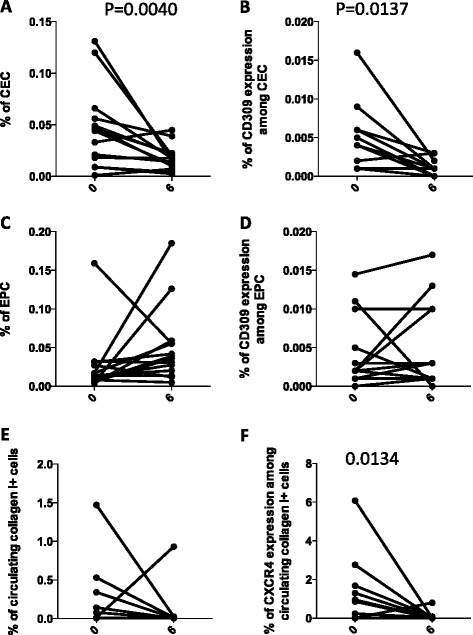
Changes in the percentages of circulating endothelial cells (*CEC*), endothelial progenitor cells (*EPC*) and circulating fibrocytes in all treated patients with idiopathic pulmonary fibrosis after 6 months of treatment. Before-and-after graphs indicate the trends of different cell populations: (**a**) the percentages of CEC; (**b**) the amount of CD309 among CEC; (**c**) the percentage of EPC; (**d**) the amount of CD309 among EPC; (**e**) circulating collagen I+ cells; and (**f**) the amount of CXCR4 among fibrocytes. *P*-values, calculated using a Wilcoxon test for paired data, only significant values are displayed

The percentages of CEC and CEC expressing CD309 were significantly decreased after 6 months of treatment (Fig. 5a, b). After 6 months, patients with IPF displayed no differences in the percentage of EPC (Fig. 5c), nor in the percentage of EPC expressing CD309 (Fig. 5d). Moreover, after 6 months of nintedanib and pirfenidone treatments, circulating fibrocytes were nearly undetectable in most patients (Fig. 5e), and circulating fibrocytes expressing CXCR4 showed a significant decrease (Fig. 5f). Likely because of the relatively low number of patients we were able to analyse, we could not find any correlation between CEC or CD309 expression, or with any clinical parameter (data not shown).

Treating patients with IPF is a major medical problem [29]. Pirfenidone attenuated the fibrocyte pool size in bleomycin-treated mouse lungs via attenuation of CCL2 and CCL12 production in vivo, and fibrocyte migration was inhibited by pirfenidone in vitro [30]. Inhibition of these cells is considered a mechanism of the anti-fibrotic action of the drug [30], and indeed pirfenidone first showed clinical amelioration in patients with IPF [31].

**Fig. 6:**
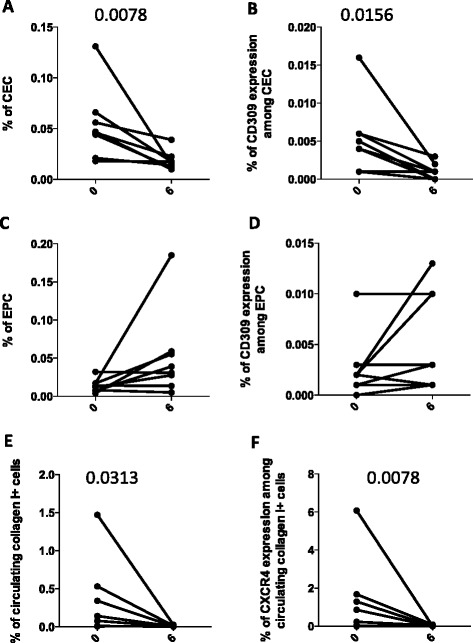
Changes in the percentages of circulating endothelial cells (*CEC*), endothelial progenitor cells (*EPC*) and circulating fibrocytes in patients with idiopathic pulmonary fibrosis treated with nintedanib after 6 months of treatment. Before-and-after graphs indicate the trend of different cell populations: (**a**) the percentages of CEC; (**b**) the amount of CD309 among CEC; (**c**) the percentage of EPC; (**d**) the amount of CD309 among EPC; (**e**) circulating collagen I+ cells; (**f**) the amount of CXCR4 among fibrocytes. *P*-values, calculated using a Wilcoxon test for paired data, only significant values are displayed

Recently, nintedanib has shown beneficial effects in patients with IPF (clinical trials *TOMORROW*, *INPULSIS 1* and *INPULSIS 2*) [32, 33]. Nintedanib was originally developed as an angiostatic factor for cancer treatments, and was approved to treat patients with lung cancer with advanced adenocarcinoma after first-line chemotherapy. Inhibition by nintedanib ultimately results in the reduced proliferation, migration and survival of fibroblasts, and potentially attenuates angiogenesis in the lung [34, 35]. Nintedanib has shown consistent anti-fibrotic and anti-inflammatory activities in bleomycin-induced pulmonary fibrosis in rodents [28, 36] and in human fibroblasts isolated from the lungs of patients with IPF, and inhibits FGF-induced, PDGF-induced, VEGF-induced profibrotic effects in human lung fibroblasts from patients with IPF [36–39]. Accordingly, in eight patients taking nintedanib, we found significant changes in CEC levels and in numbers of CEC expressing CD309, as well as in collagen I+ cells (Fig. 6). The number of patients being treated with pirfenidone was too low to allow any statistical analysis, although a similar trend was found concerning CD309 expression (data not shown).

## Conclusions

This multicentric study is the first to provide cross-sectional and longitudinal analyses of CEC and fibrocytes amongst Italian patients with IPF. Our study was performed on blood samples—we could not analyse lung tissue from patients with IPF. Indeed, the most critical obstacle to translating information obtained from molecular or cellular in vitro or ex vivo studies into clinical applications is the scarcity of lung tissue, especially in the context of a rare disease. Although some patients undergo biopsy, in most cases either lung biopsy is not indicated, or the risk associated with the procedure precludes it from being performed. Given the fact that fibrocytes might be correlated with endothelial cells during the remodelling process of fibrotic tissue, and given that drugs used in IPF may modulate the function of CEC, the aim of this study was to understand whether more accessible cells like circulating fibrocytes and endothelial cells may be used as surrogate biomarkers of disease outcome in patients with IPF treated with different drugs.

First, we investigated the phenotype of CEC and EPC and found a significant decrease in the expression of CD309 among endothelial cell populations. Thus, it is likely that the identification of such a subpopulation could be of clinical relevance. Second, we investigated the percentage of circulating collagen I+ cells, defined as fibrocytes, in patients with IPF treated with different therapies, and we found that there was no difference compared with healthy controls. The change in the expression of CXCR4 in such cells after 6 months of therapy could be indicative of a therapeutic effect, in terms of diminished homing to the lung. However, because of the relatively small number of patients we could analyse, further data are needed to clarify this aspect.

This study had some other limitations. First, we were not able to follow up the entire IPF cohort. We also could not clarify the molecular mechanism(s) by which circulating cells expressing collagen I and endothelial cells cooperate to form fibrotic foci. However, it could be hypothesized that CEC sustain the vascularization around the fibrotic foci, and thus play a pathogenic role. In conclusion, although further studies are needed to confirm that CEC and fibrocytes may be used as surrogate biomarkers of disease presence, severity, rate of progression and treatment outcome, the change in CD309 expression in endothelial cells suggests that such receptors could likely become a new target for therapies against IPF.

## Abbreviations

CEC: Circulating endothelial cells
EPC: Endothelial progenitor cells
FGF: Fibroblast growth factor
FMO: Fluorescence minus one
FSC: Forward scatter
IPF: Idiopathic pulmonary fibrosis
mAbs: Monoclonal antibodies
PDGF: Platelet-derived growth factor
VEGF: Vascular endothelial growth factor
